# PRMT1 inhibition induces differentiation of colon cancer cells

**DOI:** 10.1038/s41598-020-77028-8

**Published:** 2020-11-18

**Authors:** Alexander Plotnikov, Noga Kozer, Galit Cohen, Silvia Carvalho, Shirly Duberstein, Ofir Almog, Leonardo Javier Solmesky, Khriesto A. Shurrush, Ilana Babaev, Sima Benjamin, Shlomit Gilad, Meital Kupervaser, Yishai Levin, Michael Gershovits, Danny Ben-Avraham, Haim Michael Barr

**Affiliations:** 1 Wohl Institute for Drug Discovery, High Throughput Screening Unit, Grand Israel National Center for Personalized Medicine, Weizmann Institute of Science, Rehovot, Israel; 2 Wohl Institute for Drug Discovery, Medicinal Chemistry Unit, Grand Israel National Center for Personalized Medicine, Weizmann Institute of Science, Rehovot, Israel; 3 Crown Institute for Genomics, Grand Israel National Center for Personalized Medicine, Weizmann Institute of Science, Rehovot, Israel; 4 de Botton Institute for Proteomics, Grand Israel National Center for Personalized Medicine, Weizmann Institute of Science, Rehovot, Israel; 5 Mantoux Institute for Bioinformatics, Grand Israel National Center for Personalized Medicine, Weizmann Institute of Science, Rehovot, Israel

**Keywords:** Cancer, Chemical biology, Drug discovery, Cell biology

## Abstract

Differentiation therapy has been recently revisited as a prospective approach in cancer therapy by targeting the aberrant growth, and repairing the differentiation and cell death programs of cancer cells. However, differentiation therapy of solid tumors is a challenging issue and progress in this field is limited. We performed High Throughput Screening (HTS) using a novel dual multiplex assay to discover compounds, which induce differentiation of human colon cancer cells. Here we show that the protein arginine methyl transferase (PRMT) type 1 inhibitor, MS023, is a potent inducer of colon cancer cell differentiation with a large therapeutic window. Differentiation changes in the highly aggressive human colon cancer cell line (HT-29) were proved by proteomic and genomic approaches. Growth of HT-29 xenograft in nude mice was significantly delayed upon MS023 treatment and immunohistochemistry of tumor indicated differentiation changes. These findings may lead to development of clinically effective anti-cancer drugs based on the mechanism of cancer cell differentiation.

## Introduction

Colorectal cancer (CRC) is the third most commonly occurring cancer in men and the second most commonly occurring cancer in women, with over 1.8 million new cases in 2018^1^. The major approach for the treatments of CRC are surgery and chemotherapy, while emerging anti CRC therapies such as anti-angiogenetic therapy^2^, anti-growth factor therapy^3^, and immunotherapy^4^ were also proposed. Standard of care chemotherapy may eradicate cancer cells, but the aggressive cells can survive and resistance gradually develops. In contrast, differentiation therapy aims not to eradicate cancer bulk, but rather directs cancer cells towards inhibited proliferation and restoration of the apoptotic program, while maintaining a limited toxicity^5^. Starting from the late 1970s, there were numerous attempts to apply different kinds of chemical molecules in order to induce cancer cell differentiation^6^. Differentiation therapy has been clinically used for acute leukemia treatment^7^ and demonstrated encouraging results. In contrast to hemoproliferative disease, differentiation therapy of solid tumors is a problematic matter. Possible explanation of this is the fact that in comparison to leukemia, solid tumors have complex genetic basis and involve activation/inhibition of multiple oncogenic/differentiation pathways^8^. Several approaches of differentiation pathway targeting were reported^9^. There are usage of signaling molecules, such as retinoic acid (RA)^7^, cAMP^10^, retinoids^11^, peroxisome proliferator-activated receptor—γ (PPARγ) antagonist^12^, PTPRK-RSPO3 function-blocking antibodies^13^, inhibition of IDH1 in IDH1-mutant tumors^14^, inhibition of aurora kinase^15^, epigenetic modulators of Bromodomain-containing protein 4 (BRD4i)^16^, administration of bone morphogenetic proteins^17^, such as enhanced variant of BMP7^18^ and inhibition of histone deacetylase (HDAC). A number of HDAC inhibitors were evaluated in clinical trials and some of them such as vorinostat (SAHA), romidepsin (depsipeptide) and belinostat (PXD-101) have been approved for therapy of blood cancers by the US Food and Drug Administration (FDA)^19^. Entinostat, an advanced HDACi, was evaluated in combination with azacitidine, a demethylating agent in a phase II multi-institutional study in metastatic CRC patients. The authors reported a tolerable therapy, however without significant clinical activity^20^. Current data suggests that differentiation therapy can be an effective anti-cancer treatment approach, though discovery of new pro differentiation molecules with a higher therapeutic index is essential.

Morphologically, the differentiation changes of cancer cells can be evidenced by the formation of dome**-**like structures, a characteristic of increased water absorption, improved tight junction (TJ) function, and features reminiscent of polarized epithelial cells. Molecular differentiation biomarkers include sodium hydrogen exchanger, NHE3, and the cytoskeletal proteins villin, gelsolin^21^. The expression and localization of TJ members such as E-Cadherin and zonula occludens-1 (ZO-1) proteins indicates the status of cell differentiation^22,23^. An ubiquitous biochemical marker of cell differentiation in colon epithelial cells is the high activity of the enzyme alkaline phosphatase (ALP)^24,25^. While differentiated intestine epithelial cells express relatively large levels of the enzyme^26^, the activity of ALP in poorly differentiated colon cancer cells is known to be low or negligible^27,28^. Recently, a panel of 33 colon cancer cells lines were screened for differentiation effect of HDACi^25^ by measurement of ALP activity following treatment. The authors showed that colon cancer cell lines differ in their response to butyrate induction of ALP, where HT-29 was reported as responsive and HCT-116 as non-responsive cell line.

We recently developed a new multiplex assay applicable for HTS, where ALP activity can be measured and normalized per live cell number in the same well—CDP/CTG assay^29^. This method allows a rapid screening of compounds, which can induce differentiation of responsive colon cancer cells, using affordable and commercially available reagents. We performed a phenotypic HTS screen in HT-29 cells and discovered PRMT type 1 inhibitor, MS023, as a potent inducer of ALP activity promoting cell differentiation phenotype.

PRMT type 1 consists of 6 different enzymes: PRMT1,2,3,4,6 and 8^30^. PRMTs catalyze the transfer of methyl group from AdoMet (SAM) to guanidine-nitrogen atom that results in the methylation of arginine^31^. Arginine methylation is an important post-translational modification of proteins involved in various cellular processes such as gene regulation, RNA processing, DNA damage response, and signal transduction^32^. PRMT1 was reported to be responsible for about 90% of arginine methylation in mammalian cells^33^. Overexpression of PRMT type 1 is associated with many types of cancer. PRMT1 subtype was reported to be upregulated in colon cancer and it is associated with poor prognosis^34,35^. We confirmed PRMT1 as the main target of MS023, responsible for differentiation phenomenon, indicating a new application for PRMT1 inhibitors.

## Results

### HTS for compounds inducing differentiation of colon cancer cells

**Figure 1:**
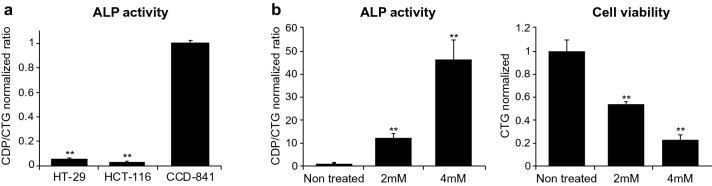
ALP activity in normal epithelial cell is much higher than in colon cancer cells; sodium butyrate (SB) increases ALP activity in colon cancer cells. (**a**) ALP activity in colon cancer cells (HT-29 and HCT-116) and normal colon epithelial cells (CCD-841). Graph represents mean ± SEM from three independent experiments. Statistical significances of ALP activity in cancer cells versus normal cells are indicated. (**b**) Effect of SB in the indicated concentrations on ALP activity and colon cancer cell (HT-29) growth, 5 days after treatment. Graphs represent mean ± SEM. from three independent experiments. Statistical significances of ALP activity and cell viability between non-treated and SB treated cells are indicated.

**Figure 2:**
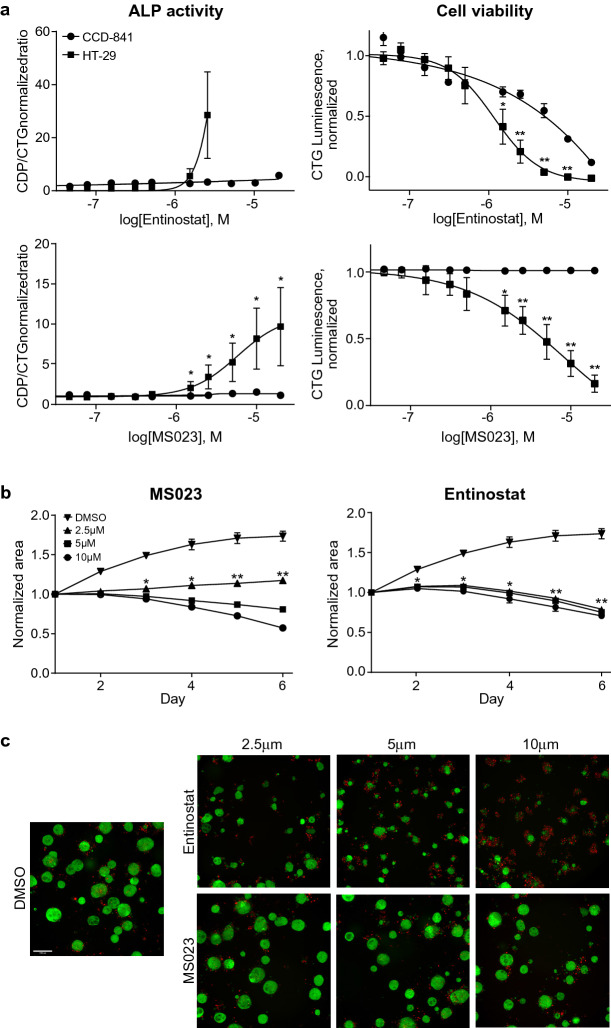
MS023 and entinostat significantly increase ALP activity and delay growth of colon cancer cells in 2D and 3D cultures. (**a**) Effect of PRMT type 1 inhibitor (MS023) and HDAC inhibitor (entinostat) on normal (CCD-841) and colon cancer (HT-29) cell growth and ALP activity in 2D culture, 5 days after treatment. The data is normalized relatively to DMSO treated cells. Graphs represent mean ± SEM from four independent experiments. Statistical significances of ALP activity in cancer cells versus normal cells are indicated. (**b**) Effect of PRMT type 1 inhibitor (MS023) and HDAC inhibitor (entinostat) on growth kinetics of HT-29 GFP expressing colon cancer spheroids (1–6 days after treatment). Graphs represent total mean spheroid area ± SEM. Statistical significances of spheroid cell growth in DMSO versus compounds treated spheroids are indicated. Graphs in (**a**) and (**b**) were made using GraphPad Prism version 8.4.3 for Windows, GraphPad Software, San Diego California USA (www.graphpad.com). (**c**) Representative pictures of the spheroids, 6 days after treatment. Green—HT-29-GFP spheroids, red—propidium iodide staining (dead cells), × 10 magnification, scale bar 200 µm.

Some cell lines were reported to be irresponsive for ALP induction by the differentiation agent sodium butyrate (SB)^25^. Therefore choosing a suitable cellular model is an essential for a HTS campaign. Two colon cancer cell lines HT-29 and HCT-116, and a normal colon epithelial cell line CCD-841 were tested for basal ALP activity. As expected, both cancer cell lines had very low ALP activity, while activity of the enzyme in CCD-841 cells was significantly higher (Fig. 1a). SB was reported as an inducer of differentiation in HT-29 cells^25^. Therefore we examined the effect of this molecule on ALP activity and found a dose dependent increase of activity together with a reduction in cell number after 5 days of treatment (Fig. 1b). Short duration (5 h) of SB treatment did not affect ALP signal or cell viability (Supplementary Fig. S1), suggesting a modulation of the phenotype rather than a direct impact on ALP enzyme activity and cell survival. Based on this data, HT-29 was considered as a suitable candidate for the HTS screening, and SB can be used as a positive control for phenotypic modulation. 5790 compounds from different chemical libraries, including 30 compounds from The Structural Genomics Consortium (SGC) Epigenetic Chemical Probe Collection, were screened at 10 µM concentration for their ability to induce ALP activity and delay growth of HT-29 cells (Z′ > 0.4). Entinostat, a well-known histone deacetylase (HDAC) inhibitor, potently induced ALP activity, however its toxicity in the primary screen was ~ 91%, leading us to de-prioritize this compound. However, due to the superior potency of entinostat over SB, it was used in further experiments as a control. Entinostat was toxic for normal cells, while its effect on colon cancer cells was stronger (Growth Inhibition of 50% (GI50) was 8.3 µM and 1.2 µM, respectively). The most pronounced ALP-inducing hit was PRMT type 1 inhibitor MS023. Figure 2a shows that MS023 moderately increased ALP activity in comparison to entinostat (e.g. 5-fold increase versus about 30-fold increase respectively at 2.5 µM). Effective concentration of 50% (EC50) of MS023 was 5.8 µM, and it significantly delays HT-29 cell proliferation (GI50 was 2.3 µM). Importantly, MS023 had no effect on either ALP activity or proliferation of CCD-841 cells. Interestingly, MS023 reduced cell growth in four colon cancer cell lines, previously reported as irresponsive to SB treatment^25^: HCT-116, Colo-205, SW-620, and HCT-15 (Table 1). This suggests that MS023 effect was not cell line specific^36^.

**Table 1:** Fold increase of ALP activity and percent of viable cells in indicated colon cancer cell lines treated with 20 µM of MS023 for 5 days, compared with DMSO treated control, mean ± SEM.

Cell line	ALP activity, fold ± SE	Cells, percent ± SE
HT-29	7.9 ± 0.14	38 ± 0.3
HCT-116	0.9 ± 0.02	53 ± 0.5
HCT-15	1.4 ± 0.15	46 ± 0.4
Colo-205	2.6 ± 0.12	33 ± 0.3
SW-620	1.5 ± 0.26	43 ± 0.4

Three dimensional cultures (3D, tumor spheroids and organoids) are believed to be an improved in vitro model since they represent a more physiologically relevant pattern of cell growth. In comparison to monolayer, 3D culture may demonstrate profound differences in proliferation, differentiation, morphology and other cellular functions^37^. HT-29 rapidly forms spheroids in an alginate-based matrix. Following formation of established spheroids (5 days), MS023, entinostat or vehicle (DMSO) were added and growth was monitored during 6 additional days. In order to detect dead cells, we performed propidium iodide (PI) staining of the spheroids shortly before imaging (Fig. 2b,c). While entinostat rapidly induced cytotoxic effect, MS023 showed reduced growth, but low toxicity. Thus, PRMT type 1 inhibition induces effects associated with cell differentiation in both monolayer and 3D culture.

### Target validation: inhibition of PRMT1 mediates a differentiated phenotype

**Figure 3:**
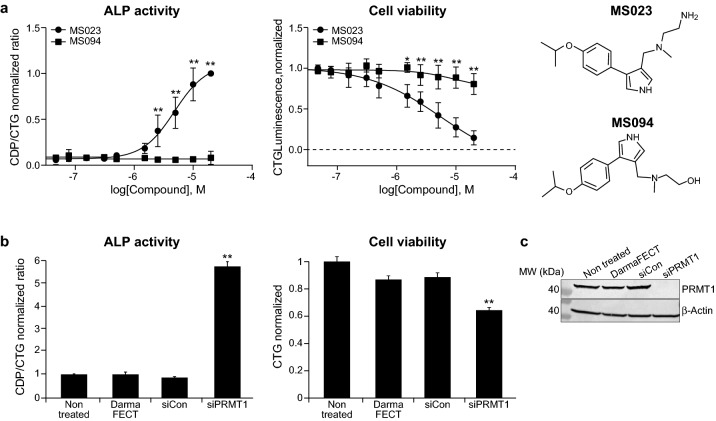
PRMT1 inhibition/knock down mediates differentiation phenotype in colon cancer cells. (**a**) Effect of PRMT type 1 inhibitor (MS023) and its inactive analog (MS094) on colon cancer cell (HT-29) growth and ALP activity, 5 days after treatment. The data is normalized relatively to DMSO treated cells. Graphs represent mean ± SEM from three independent experiments. Graph was made using GraphPad Prism version 8.4.3 for Windows, GraphPad Software, San Diego California USA (www.graphpad.com). Statistical significances of ALP activity and cell viability between MS023 and MS094 treated cells are indicated. Chemical structures of the compounds are shown on the right side of the graph. (**b**) Effect of PRMT1 knock down on HT-29 growth and ALP activity, 5 days after treatment. Groups: non treated cells, DharmaFECT – cells treated with transfection reagent alone, siCon, cell transfected with relevant control siRNA, siPRMT1—cells transfected with siPRMT1. Graphs represent mean ± SEM from three independent experiments. Statistical significance of ALP activity and cell viability between siPRMT1 and others groups is indicated. (**c**) Western blot analysis of PRMT1 expression in the indicated groups of HT-29 cells, 5 days after transfection. Full-length blots are presented in Supplementary Fig. S2.

Due to the well-defined target, selectivity of a chemical probe, inactive but structurally similar compounds are extremely useful. MS094 is a close analog of MS023, and was reported to be inactive against PRMT type 1 in biochemical assays^38^. Indeed, MS094 failed to induce ALP activity in HT-29 cells and did not affect cell proliferation (Fig. 3a), confirming that engagement of PRMT type 1 is likely driving the observed phenotype. MS023 was reported to be a potent inhibitor of PRMT1, 3, 4, 6 and 8^38^. Chemical probes of PRMT3, 4 and 6 (SGC707, TP-064 and MS049) are present in the Epigenetic Chemical Probe Collection (SGC) library, and they were unable to induce a differentiated phenotype in colon cancer cells, while PRMT8 is a brain specific protein^39^. Considering the selectivity profile of the epigenetic probe library, it is likely that PRMT1 is the sole target driving the probe induced differentiation phenotype. In order to prove this hypothesis, we employed genetic ablation of PRMT1 using siRNA. As shown in Fig. 3b,c we observed a significant elevation of ALP activity and growth delay in siPRMT1 treated HT-29 cells compared to controls (Full-length blots from Fig. 3c are shown in Supplementary Fig. S2).

### Target validation: cellular thermal shift assay (CETSA) validate PRMT1 as a target of MS023.

**Figure 4:**
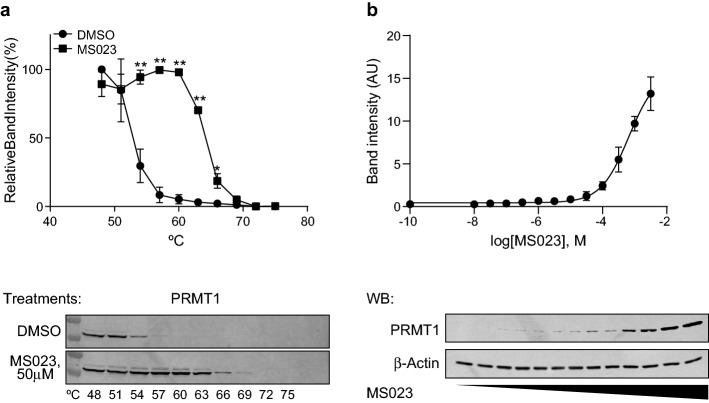
MS023 stabilizes PRMT1. (**a**) Cellular thermal shift assay of MS023 in HT-29 cells. Graph represents relative PRMT1 band intensities of the indicated samples ± SEM from three independent experiments. Representative PRMT1 Western blots of each sample are shown. Statistical significances between MS023 and DMSO are indicated. The analysis was performed simultaneously in the MS023 and DMSO samples with same time of the exposure. Full-length blots are presented in Supplementary Fig. S3. (**b**) Isothermal (at 63 °C) dose response fingerprints in HT-29 cells. Graph represents relative PRMT1 band intensities of the indicated samples ± SEM from three independent experiments. Graphs in **a** and **b** were made using GraphPad Prism version 8.4.3 for Windows, GraphPad Software, San Diego California USA (www.graphpad.com). Representative PRMT1 and actin Western blots of each sample are shown. Full-length blots are presented in Supplementary Fig. S4.

CETSA is a well-established assay for target validation and evaluation of physiological binding conditions inside of cells^40^. First, stabilization of PRMT1 by MS023 was analyzed in living HT-29 cells using temperature gradient. We found a significant PRMT1 stability shift compared with DMSO treated control (about 12 °C) (Fig. 4a). While PRMT1 band completely disappeared at the temperature of 60 °C in the group of DMSO treated cells, similar effect in MS023 treated group was observed in the temperature of 72 °C. In order to more quantitatively understand the MS023 binding affinity, we performed isothermal dose response fingerprint analysis based CETSA. Figure 4b shows MS023 dose dependent stabilization of PRMT1. Full-length blots from Fig. 4a,b are shown in Supplementary Figs. S3 and S4 respectively).

### Phenotype validation: PRMT1 inhibition drives colon cancer cell differentiation

**Figure 5:**
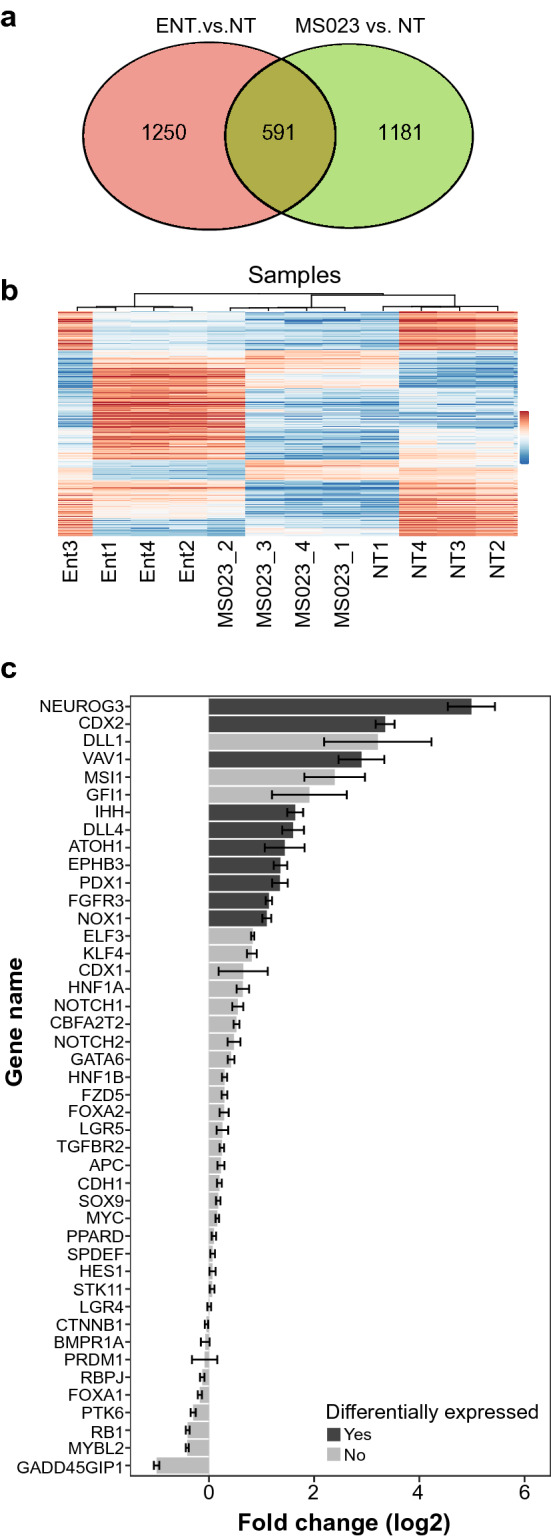
MS023 treatment drives colon cancer cell differentiation: genomic analysis. (**a**) Overlap between differentially expressed genes, 5 days after entinostat (1.5 µM) or MS023 (5 µM) treatments (n = 4/group). (b) Heat map of the differential expressed genes. The values are counts on normalized log2 scale. (**c**) Log2 fold changes in expression of genes, which are positively involved in the differentiation of normal intestinal cells. Comparison of samples derived from HT-29 cells treated with MS023 (5 µM, n = 4) and DMSO (n = 4), 5 days after treatment, mean ± SEM. Bold grey color columns represent differentially expressed genes. Statistical significances (pV) of all differentially expressed genes between MS023 and DMSO treated samples are < 0.001.

Broad changes in gene expression patterns are expected after treatments with differentiation inducing chemical probes. Since both entinostat and MS023 treatments drove an increase in ALP activity, the associated gene-expression profile of each phenotype was examined. Figure 5a shows that in spite of some overlap, the majority of genes changed differently. Clustering of genes shown in Fig. 5b demonstrates significant distinction between all treated groups. Focusing on genes with documented involvement in positive regulation of differentiation status of normal intestinal cells^41^ showed that MS023 treatment resulted in elevation of more than 65% of them, while ~ 23% were differentially expressed, and none of the candidate genes was significantly decreased (Fig. 5c).

**Figure 6:**
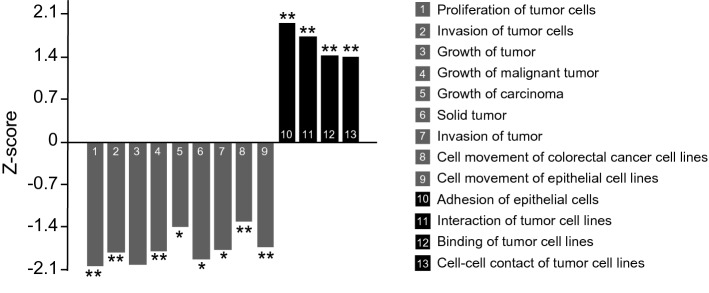
MS023 treatment drives colon cancer cell differentiation: proteomic analysis. Graph represents changes in pathways associated with cancer progression (grey columns) and cell differentiation (black columns). Comparison of MS023 treated (5 µM, n = 8) and DMSO treated (n = 8) samples of HT-29 cells, 5 days after treatment. Statistical significances of the indicated pathways changes upon MS023 treatment are indicated.

Next, we performed proteomic analysis of non-treated and MS023 treated samples. Our focus was on changes in the expression of proteins, which are considerably involved in either tumor progression or cell differentiation. As shown in Fig. 6, the majority of the proteins involved in tumor progression were significantly down regulated. Importantly, such changes were observed not only in tumor proliferation pathways, but also in cancer cell motility and invasiveness pathways, suggesting that PRMT1 inhibition might prevent metastatic progression. In contrast, expression of proteins that characterize less aggressive tumor or even normal tissue was significantly upregulated. This trend was even more evident in the proteins responsible for cell–cell contact and cellular adhesion. Yet, expression of ALP was increased ~ 20 fold in MS023 treated samples, proving the efficacy of our assay in the measurement of ALP activity, and strongly supporting the usage of ALP as a differentiation marker of colon cancer cells. Full list of the differentially changed proteins and list of the proteins associated with particular pathways are presented in the Supplementary Tables S1 and S2.

**Figure 7:**
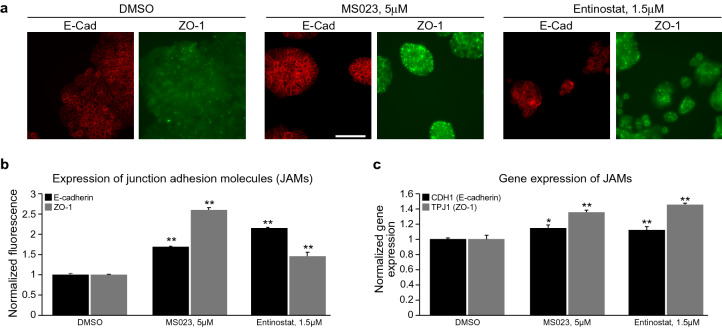
MS023 and entinostat treatment increase expression of junction adhesion molecules (JAMs) E-cadherin and ZO-1 and their genes in HT-29 colon cancer cells. (**a**) Representative pictures of HT-29 cells treated with indicated compounds and stained for E-cadherin (red) and ZO-1 (green), × 40magnification, scale bar 100 µM. (**b**) Graph represents mean ± SEM of normalized fluorescence derived from pictures presented in “**a**”. Statistical significances of JAMs induction upon MS023 and entinostat treatments are indicated. (**c**) Effect of MS023 and entinostat treatments on changes in the expression of E-cadherin and ZO-1 genes in HT-29 cells. Graph represents log2 change of normalized fluorescence ± SEM in the cells treated with indicated compounds in comparison to DMSO treated cells. Statistical significances of gene induction of JAMs upon MS023 and entinostat treatments are indicated.

Intestinal epithelium and CRC cells differ in the expression and intracellular localization of Junctional Adhesion Molecules (JAMs)^20,21^. We analyzed both, gene and protein expression of JAMs (namely E-Cadherin and ZO-1). Immunostaining of cells allowed us to monitor also intracellular localization of JAMs. MS023 and entinostat treatments slightly increased JAM gene and protein expression and causing corrected intracellular localization in HT-29 cells (Fig. 7a–c). Intriguingly, HCT-116 colon cancer cell line, which was reported as not responder to SB treatment, as suggested by the lack of induction of ALP activity^25^, still demonstrated a slight increase in E-Cadherin, while ZO-1 pattern was similar to HT-29 cells (Supplementary Fig. S5). This implies that the differentiation phenomenon could be a more general effect in CRC.

### Differentiation therapy is effective against human colon cancer in mouse xenograft model

**Figure 8:**
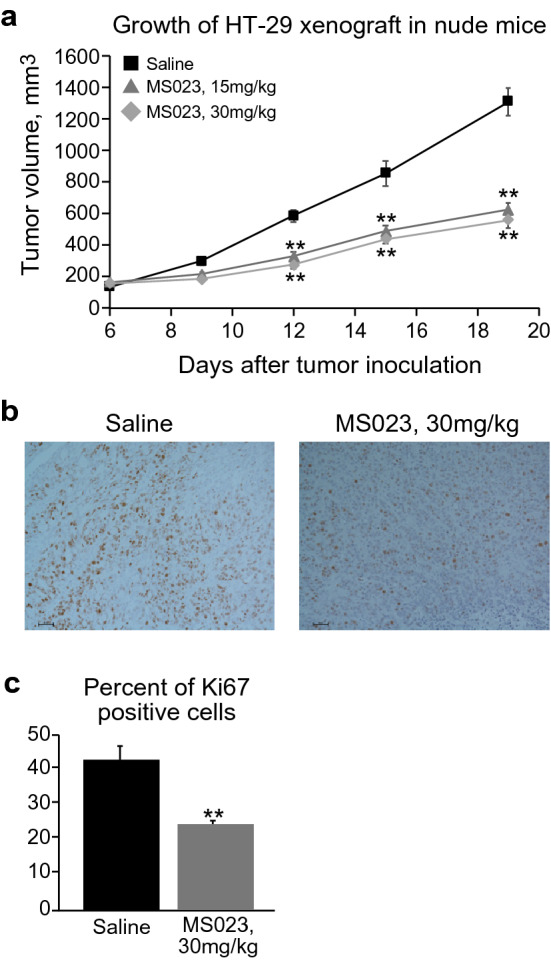
MS023 significantly inhibits HT-29 xenograft growth and delays cancer cell proliferation in-vivo. (**a**) Kinetics of HT-29 xenograft growth in nude mice (n = 5/group). Data represented as tumor volume means ± SEM. Grey bright diamonds—MS023, 30 mg/kg; grey bold tringles – MS023, 15 mg/kg; black squares—saline control. Statistical significances of tumor volumes between saline treated and MS023 treated groups are indicated. (**b**) Representative images of Ki67—DAB stained HT-29 xenograft taken from saline (n = 3) and MS023 treated mice (n = 5), scale bar 50 µm. (**c**) Graph represents present of Ki67 positive cells ± SEM. Statistical significance of Ki67 changes upon MS023 treatment is indicated.

MS023 efficacy in-vivo was examined using HT-29 xenograft model in nude mice. In order to calculate desirable frequency of MS023 injection, a preliminary experiment was performed, where cell growth medium containing MS023 was replaced 5 days later with a fresh one containing or not a second dose of MS023. The absence of MS023 led to restoration of the proliferative potential, while addition of a second dose kept the cells in low-proliferating status (data not shown). Therefore, the animals received 2 doses of the compound/week. The treatment started when tumors reached 6–7 mm diameter. MS023 treatment significantly delayed tumor growth (Fig. 8a) without any signs of toxicity. Animal organs (spleens, lungs, livers) were taken and inspected postmortem, and were found similar to those of normal animals, suggesting absence of systemic toxicity.

Reduced proliferation is a characteristic of cancer cells undergoing differentiation. A well-known marker of cell proliferative status is Ki67, proliferation-associated nuclear protein, which is detected exclusively in dividing cells in any cycle of cell division. Its expression strongly correlates with clinical stage of tumor and metastatic burden^42^. In accordance to a scale proposed by Jonat et al.^43^, tumor proliferation status assessed by Ki67 index is divided into 3 different categories, where tumor containing of more than 30% of Ki67 expressing cells is considered as highly proliferating, 16–30% as intermediate proliferating, and 15% or less than as low proliferating. Immunohistochemistry staining of HT-29 xenografts with Ki67 antibodies was performed. Figures 7b, c shows significant decrease of Ki67 labeling index from 34.6 ± 2.7% (highly proliferating) to 19.1 ± 1.9% (intermediate proliferating).

## Discussion

While the genome-driven targeted drug discovery model was the accepted path for most industrial campaigns of the last 20 years, the number of approved drugs discovered through phenotypic screens was found to be statistically greater than those discovered through the molecular target-based approach^44^. Therefore, in the last decade, phenotypic screens have been reestablished as a main methodology in drug discovery. Although it is a challenging path, a compound with a validated target from a relevant phenotypic assay seems to be better fit for probable progression towards a drug. Phenotypic assays are frequently costly and difficult to automate, thus impacting the number of compounds that can be screened, making the choice of chemical matter of primary importance to the success of the screen. The use of bioactive compounds and chemical probes can bridge this gap, as they will sample druggable targets, and provide a focus for expansion of the chemotypes around that target.

Cancer cell phenotype is significantly different from that of normal cells. There are some “hallmarks of cancer”, well described by Hanahan and Weinberg^45^. The key cancer hallmark is uncontrolled cell proliferation, therefore inhibition of proliferation, induction of apoptosis and killing of cancer cells were and remain the main goals in anticancer drug discovery^46^. Unfortunately, the majority of drug candidates discovered by such methods exhibit little if any therapeutic benefit in clinical trials^47^, mostly due to unexpected toxicity^48^. In contrast, differentiation therapy is potentially less aggressive if used as a single agent or in combination with lower-dose of cytotoxic drugs. Given that majority of cancer mortality is caused due to metastases, a decrease in metastatic potential by differentiation might have a great benefit as a therapy. Yet, the majority of differentiation focused therapies were performed using leukemia models, while solid tumors have been less investigated^6^.

Epigenetic alterations are common in cancer and can promote various features such as malignant cell transformation, development of the disease, metastatic procession and resistance to chemotherapy^49^. Epigenetic modulation, via HDAC inhibitors, has been reported as a means for inducing therapeutic differentiation^21^. It was confirmed in the screen by the differentiation activity of sodium butyrate and entinostat. Whereas considered to be a highly selective HDAC type 1 inhibitor, entinostat demonstrated high toxicity against both cancer and normal colon cells. One can speculate that such low therapeutic window can be one of the reasons for failure of entinostat in clinical studies^20^. In contrast, PRMT type 1 inhibitor, MS023, significantly delayed colon cancer cell/spheroids growth without any effect on growth of normal cells (Fig. 2), and thus was selected as a probe with the potential to modulate the CRC phenotype. Since MS023 can inhibit a few different PRMTs type 1 in-vitro^38^, it was important to discover a particular PRMT subtype, which is responsible for the induction of differentiation phenomenon in cellular models. Using both siRNA and CETSA approaches, it was shown that: (1) PRMT1, a member of PRMT type 1, is a target for MS023, which was able to bind and stabilize it in living HT-29 cells (Fig. 4a,b); (2) inhibition of PRMT1 plays the main role in the induction of HT-29 cell differentiation by MS023 (Fig. 3b).

PRMT type 1 has attracted a close attention of pharmaceutical industry as a promising anticancer target. GlaxoSmithKline (GSK) performed intensive studies across 249 cancer cell lines, representing 12 tumor types^50^. Some of the cancer lineages especially blood cancers showed a strong cytotoxicity profile, while such effect in solid tumors was less pronounced. It is remarkable that in their studies the IG50 in the majority of colon cancer cell lines was reported to be between 1 and 10 µM, which is similar to IG50 observed in our experiments (2.3 µM, Fig. 2a).

Model in vitro cell culture systems with limited predictivity can affect the ability to succeed in a clinical trial. For example, 2D monolayer cultures significantly differ from 3D spheroids in many aspects such as physical pressure; cell/cell contact; cell shapes, orientation and polarity; nutrition and oxygenation; paracrine status, etc. For example, Karlsonn et al. reported that colon cancer (HCT-116) spheroids were significantly less sensitive than cells growing in 2D monolayer^51^ to four frequently used CRC chemotherapeutic drugs. Therefore, the effect of MS023 and entinostat in 3D culture of HT-29 cells was examined. This assay revealed similar effect of these compounds on cell growth in both 2D and 3D models. Importantly, MS023 and entinostat were added when spheroids were already formed, implying that the growth delay was not preventing spheroid formation and would more accurately represent a clinical context of treatment. In both models, entinostat had strong cytotoxic effect, while no remarkable toxicity was observed in MS023 treated cells/spheroids (Fig. 2b,c).

In order to further characterize the pro-differentiation phenotype, gene expression in non-treated HT-29 cells and cells treated with either entinostat or MS023 was compared. It was found that the gene expression profile in treated groups was significantly different (Fig. 5a,b). Since downstream targets of HDAC and PRMT type 1 are different, the mechanisms of differentiation mediated by inhibition of PRMT type 1 can be different from those mediated by inhibition of HDAC, suggesting a potential for combination therapy. MS023 treatment resulted in upregulation of many genes involved in intestinal cell differentiation (about 23% of all such genes were differentially expressed) (Fig. 5c). For example, the expression of Neurogenin 3, a gene that stimulates a program of terminal enteroendocrine cell development^52^, and is critical for epithelial cell functionality^53^ was elevated about 25-fold. The expression of CDX2 gene, a master regulator of intestinal phenotype that plays a tumor-suppressive role in colon cancer expression^54^ was increased ~ tenfold. Another important gene upregulated under MS023 treatment is a regulator of enterocyte/colonocyte differentiation named ATOH1 (Math1/Hath1). In colorectal cancer, ATOH1 is a tumor suppressor and is silenced^55^.

The fact that MS023 treatment resulted in less aggressive phenotype of colon cancer cells was further proved by deep analysis of protein expression profile. In this study, we focused on changes in proteins, which can characterize tumor phenotype. HT-29 is aggressive, metastatic colon cancer cell line^56^. MS023 treatment significantly changed its phenotype toward less aggressive status. The proteins involved in nine critical pathways related to tumor aggressiveness were significantly down regulated. Importantly, we identified the down regulation of the proteins responsible for cancer cell movement and invasive potential. Moreover, MS023 treated cells showed increasing expression of cell–cell contact and adhesion proteins, which is a hallmark of less aggressive and more differentiated cells.

Junctional Adhesion Molecules (JAMs) are important for proper functions of cell-to-cell tight junctions. They regulate epithelial and endothelial cell adhesion and polarity. Abnormality in JAMs are involved in the Epithelial-Mesenchymal Transition (EMT), a process that plays a crucial role in the invasiveness and metastasis of various cancers^57^. E-cadherin, encoded by the CDH1 gene, is a transmembrane protein critical for the normal function of epithelial cell–cell junction, and neoplastic processes in the colon are characterized by loss and/or aberrant localization of E-cadherin. This results in two main consequences: (1) disruption of cell–cell contacts that promotes metastatic process; (2) dissociation of E-cadherin-β-catenin complex. Ultimately, this results in loss of differentiation and activation of β-catenin mediated cell proliferation^22^. ZO-1, is a member of the Zonula occludens protein family which consists of scaffold-forming intracellular proteins located between the transmembrane proteins and the actin cytoskeleton. They bind actin, occludin, and claudins and regulate the assembly of cellular junctions. In differentiated normal epithelial cells, ZO-1 mostly localizes at the cell–cell adhesion membrane complexes. Similar to β-catenin, ZO-1 detaches from the membrane complexes in cancer cells, accumulates in the cytoplasm, and eventually translocates to the nucleus to promote proliferation and invasiveness^58^. Both gene and protein expression of JAMs as well as their intracellular localization were analyzed. MS023 and entinostat treatments slightly increased JAM gene and protein expression and corrected intracellular localization (Fig. 7). Intriguingly, approximately half of colon cancer cell lines, including HCT-116, did not respond to the treatment with known differentiation agent (SB) by increasing of ALP activity^25^. However, HCT-116 demonstrated JAMs pattern similar to HT-29 cells (Fig. 6), implying that the differentiation phenomenon could be a more general effect in CRC.

MS023 is a well characterized molecular probe^38^, however its effects were not examined in animal models. Effectiveness of a molecule in whole organisms does not always correlated with that in vitro*.* Absorption, distribution, metabolism, and excretion (ADME) properties of a potential drug as well as its toxicity can be non-appropriated that might result in failure of such compounds in animal studies. We observed significant delay of HT-29 xenograft growth, while there was no statistical difference in tumor volume between two groups treated with MS023 (15 and 30 mg/kg). We hypothesize that we reached a dose plateau, and increasing the frequency of injections rather dose augmentation, will strengthen anti-tumor effect of MS023. Given that numerous cytotoxic drugs lead to tumor growth delay, it was important to examine differentiation status of tumor xenograft. Proliferation index is an established marker of tumor aggressiveness^39,41^. Its measurement allowed us to conclude that MS023 treatment was able to significantly reduce the proliferative potential of HT-29 xenograft, suggesting differentiation changes in the treated tumors. Our data shows that MS023 was able to reduce tumor growth and proliferation in vivo through differentiation of malignant cells*,* demonstrating a tractable model of pharmacological manipulation of colon cancer differentiation, with clinical implications.

## Methods

### Cells culture and handling

HT-29, HCT-116, HCT-15, SW-620, Colo-205 colon cancer and CCD-841 normal colon epithelial cell lines were purchased from ATCC. GFP expressing HT-29 cells were kindly provided by Dr. Z. Elazar lab, the Weizmann Institute of Science, Rehovot, Israel. Cancer cells were grown in Roswell Park Memorial Institute growth medium (RPMI-1640, Biological Industries Israel, # 01-101-1A) and normal cells in Eagle's Minimum Essential Medium (EMEM, Sigma-Aldrich, #M22790) mediums supplemented with 10% heat inactivated Fetal Bovine Serum (FBS), 1 mM l-glutamine and 1% penicillin/streptomycin solution (all from Biological Industries). Exclusion of Mycoplasma contamination was monitored and conducted by test with Mycoalert kit (LONZA, #BELT07-218).

### HTS equipment

Screen and hit validation experiments were performed using the HTS equipment: Echo555 Acoustic transfer system (Labcyte, Germany), Combi MultiDrop (Thermo Fischer Scientific), Washer Dispenser II (GNF, San Diego, CA, USA), EL406 Microplate Washer Dispenser (BioTek, Winooski, VT, USA), Bravo Automated Liquid Handling (Agilent, Santa Clara, CA, USA). Luminescence signals were measured by luminescence module of PheraStar FS plate reader (BMG Labtech, Ortenberg, Germany). Acumen laser scanner (TTP Labtech, Hertfordshire, United Kingdom) and ImagExpress Micro XL high content microscope (Molecular Devices, Sunnyvale, CA, USA) were used to acquire and analyze images.

### Screening procedure and CDP/CTG multiplex luminescence assay

5760 compounds from Selleck Chemicals Bioactive, MEGxp Pure natural compounds (Analyticon), Drug-Like Set (DLS, Enamine) and whole SGC Epigenetic Chemical Probe Collection chemical libraries (30 compounds) were screened using HT-29 cells by previously described multiplex method for detection and normalization of ALP level in cells^29^. Briefly, 250 cells/well were plated in 50 µl of growth medium into white/white 384-well TC plate (Greiner, #781080) and treated with either compounds or controls (sodium butyrate, DMSO). After 5 days cells were washed 4 times, lysed, and two consequent luminescent signals (CDP for alkaline phosphatase activity and CTG for cell viability) were measured. Similar procedure was used in all follow up experiments, where ALP activity and cell viability were measured.

### Data analysis, normalization and statistics

Screening and hit validation data were plotted and analyzed using GeneData 12 and 15 (Basel, Switzerland), and Collaborative Drug Discover (CDD) softwares (Cambridge, United Kingdom). Other statistical significances were evaluated by two tailed t test, *p < 0.05, **p < 0.01. ALP activity and cell viability data were normalized to DMSO treated controls. Genomic and proteomic methods for data analysis described in the related sections below.

### Cell staining and image processing

250 cells/well were plated in 50 µl of growth medium into black/transparent 384-well TC plate (Greiner, #781091) and treated with appropriated compounds/controls. After 5 days cells were fixed with 4% PFA (Santa Cruz, #30525-89-4), permeabilazed with 0.2% Triton in 2% BSA (both from Sigma Aldrich) and stained with appropriated antibodies (anti-E-Cadherin-Alexa Fluor 647 conjugated, Abcam, #ab194982; anti-ZO1, BD, #610966; Alexa Fluor 488 goat anti-mouse IgG, Invitrogen, #A11001) in accordance to the manufacture protocols. Images were taken using ImageXpressMicro confocal microscope (Molecular Devices, Sunnyvale, CA, USA) using × 20 lens with appropriate filters. Image analysis was done using MetaXpress in house developed algorithm (Custom Module Editor).

### Spheroids growth in 3D culture

Green Fluorescent Protein (GFP) expressed HT-29 cells (500 cells/well) were plated in 50 µl of growth medium supplemented with SpheraMax synthetic polymer (provided by Nissan Industries, Japan) into repellent surface plate (Greiner, #781970). Compounds were added to the formed spheroids 5 days after cell plating. In compound treated spheroids, DMSO was adjusted to the maximum concentration of 0.1%. Spheroid growth was monitored during additional 6 days and then cells were stained with propidium iodide (Sigma Aldrich, #P4864). Images were taken and analyzed as described above.

### PRMT 1 knock down

HT-29 cells were plated into 6-well plate (35,000/well) and into 384-well plate (250/well) in antibiotic free medium. Next day cells were transfected with either non-relevant siRNA, or siPRMT1 (both from Dharmacon, # L-010102-00-0005 and D-001810-10-05 respectively) using DharmaFECT 1 transfection reagent (T-2001-01) in accordance to manufacture protocol. 5 days later cells were subjected to CDP/CTG multiplex assay (384-well plate), and Western Blotting (6-well plate) using anti-PRMT1 and β-actin (C4) antibodies (Santa Cruz, Sc-1666963; Sc-47778).

### Lysate preparation and immunoblotting analyses

For sample preparation, cells were lysed in RIPA buffer (Sigma Aldrich, #R0278) mixed with protease inhibitor cocktail (1:100 dilution, Sigma Aldrich, #118735). Cells were then centrifuged at 14,000 × rpm for 20 min at 4 °C. The supernatants were collected and transferred to a fresh tube. Samples were boiled at 70 °C for 10 min in NuPage LDS sample buffer (Thermo Fischer Scientific, #NP0007) and 1,4-Dithiothreitol (DTT, Sigma Aldrich, #D9779). Proteins were separated on Novex™ 4–12% Tris/Glycine gel (Thermo Fischer Scientific, #XP04200). Gels were then transferred using the iBlot™ 2 Transfer Stacks, nitrocellulose, regular size (Thermo Fischer Scientific, #IB23001). After transfer, membranes were blocked for 1 h in 1 × Tris-buffered saline (Sigma Aldrich, #T5912) with 0.1% Tween 20 (Sigma Aldrich, # P1379) and 5% (wt/vol) BSA (Sigma Aldrich, # A7906) followed by overnight incubation of the respective antibodies at 4 °C. After TBST washing steps, membranes were incubated for 1 h at room temperature with HRP-conjugated anti-mouse secondary antibodies (1:5000 dilution, Thermo Fisher Scientific, ##G21040). Membranes were then developed with the ECL Prime Western Blotting Detection Reagent (GE Healthcare, # RPN2232), signal was measured and band intensities quantified using an iBright system (Thermo Fisher Scientific).

### Cellular thermal shift assay (CETSA)

The CETSA assay was performed as described in Miettinen and Bjorklund^59^ with some modifications. Briefly, HT-29 cells were trypsinized, washed in PBS and then suspended in PBS containing protease inhibitor cocktail. The suspended cells were then divided into two Eppendorf tubes and were treated with either MS023 (50 µM) or DMSO for 1 h at 37 °C with shaking. Following treatment, each sample was then divided into PCR tubes (100 uL/tube) and subjected to a temperature gradient (ranging from 48 to 72 °C) for 3 min. Cell lysates were obtained by 3-cycles of freeze–thaw using liquid nitrogen and a thermo block set to 25 °C. Samples were then centrifuged at 15,000 rpm at 4 °C for 20 min and were subsequently analyzed by Western Blot. The isothermal dose response fingerprint (ITDRF) experiments were done using a constant temperature of 63 °C. Band intensities were normalized to the highest concentration and β-Actin levels. Analysis of the results were done in accordance to Jafari et al.^40^ using GraphPad Prism software.

### Compounds quality control

MS023 and MS024 in DMSO (stock of 10 mM, 0.5 µl/well) were added to 384-well plate and then dissolved in 50uL of water: ACN (7:3), following centrifugation for 30 s. LC Method: Gradient solvent A (100%) to solvent B (100%) 4 min, hold solvent B 1 min. Solvent A Name: 90:10 Water: ACN 0.05%, Solvent B Name: ACN 0.05% FA. Flow Ramp Rate: 0.45 min, Run Time: 5 min, Injection Volume: 7µL. The analysis was performed using “Waters LCMS ACQ-SQD2#LCA695” instrument. Instrument Read backs: Capillary (kV) 1.47, Corona (uA) 1.74, Corona (kV) 3.1, ES Cone (V) − 1.71, APCI Cone (V) 23.69, Extractor (V) 4.64, Source Temperature (°C) 149, Desolvation Temperature (°C) 350, Cone Gas Flow (L/Hr) 8, Desolvation Gas Flow (L/Hr) 645. QSM.

### Genomics sample preparation

HT-29 cells were plated in 6-well plates (30,000 cells/well) in 1.5 ml of regular growth medium. 2 h later cells were treated with DMSO, entinostat (1.5 µM) or MS023 (5 µM). After 5 days RNA was eluted using RNeasy Mini Kit (Qiagen, #74104) according to the manufacture protocol. Libraries were prepared using the INCPM-mRNA-seq. Briefly, the polyA fraction (mRNA) was purified from 500 ng of total RNA following by fragmentation and the generation of double-stranded cDNA. Then, end repair, A base addition, adapter ligation and PCR amplification steps were performed. Libraries were evaluated by Qubit (Thermo Fisher scientific) and TapeStation (Agilent). Sequencing libraries were constructed with barcodes to allow multiplexing of 12 samples in 1 lane. Around 22–26 million single-end 60-bp reads were sequenced per sample on Illumina HiSeq 2500 V4 instrument (described in^36^).

### Proteomic sample preparation

HT-29 cells were plated in 10-well plates (120,000 cells/well) in 10 ml of regular growth medium. 2 h later cells were treated with either DMSO or MS023 (5 µM). After 5 days cells were trypsinazed, washed with PBS, and pellets were stored in − 80 °C until processing. Frozen HT-29 cell pellets were lysed in 5% Sodium dodecyl sulfate (SDS, #L3771) in 50 mM Tris pH 7.4 and 100 µg of total protein were taken for the tryptic digest. The samples volume was adjusted to 50 µl with 50 mM ammonium bicarbonate, digested with trypsin using S-trap (Protifi, Huntington NY, USA)^60^ according to the manufacturer’s instructions, vacuum dried and stored in − 80 °C prior to fractionation. All chemicals were from Sigma Aldrich, St. Louis MO, USA, unless stated otherwise.

### Liquid chromatography

Liquid chromatography was performed as previously described^60^ with the following changes: ULC/MS grade solvents were used for all chromatographic steps. Each sample was fractionated using high pH reversed phase followed by low pH reversed phase separation. 100 µg digested protein was loaded using high Performance Liquid Chromatography (Acquity H Class Bio, Waters, Milford MA, USA). Mobile phase was: (A) 20 mM ammonium formate pH 10.0, (B) acetonitrile. Peptides were separated on an XBridge C18 column (XBridgeC18, 2.5 um, 3.0 × 150mm (186006710) Waters) at 45 °C, 300 µl/min flow using the following gradient: 3% B for 2 min, linear gradient to 40% B in 50 min, 5 min to 95% B, maintained at 95% B for 5 min and then back to initial conditions. Peptides were fractionated into 15 fractions. The fractions were then pooled: 1 was combined with 2, 3, 13, 14 and 15; 4 with 9; 5 with 10; 6 with 11; 8 with 12; while 7 was kept separately. Each fraction was vacuum dried, then reconstituted in 50 µl of 97:3 acetonitrile: water + 0.1% formic acid. Each pooled fraction was loaded using split-less nano-Ultra Performance Liquid Chromatography (10 kpsi nanoAcquity; Waters, Milford, MA, USA). The mobile phase was: (A) H_2_O + 0.1% formic acid and (B) acetonitrile + 0.1% formic acid. Desalting of the samples was performed online using a reversed-phase Symmetry C18 trapping column (180 µm internal diameter, 20 mm length, 5 µm particle size; Waters). The peptides were then separated using a T3 HSS nano-column (75 µm internal diameter, 250 mm length, 1.8 µm particle size; Waters) at 0.35 µl/min. Peptides were eluted from the column into the mass spectrometer using the following gradient: 4–27% B in 105 min, 27–90% B in 5 min, maintained at 90% for 5 min and then back to initial conditions.

### Mass spectrometry

Mass spectrometry analysis was performed as previously described^60^. Briefly, the nanoUPLC was coupled online through a nanoESI emitter (10 μm tip; New Objective; Woburn, MA, USA) to a quadrupole orbitrap mass spectrometer (Q Exactive HFX, Thermo Scientific) using a FlexIon nanospray apparatus (Proxeon). Data was acquired in data dependent acquisition (DDA) mode, using a Top10 method. MS1 resolution was set to 120,000 (at 400 m/z), mass range of 375–1650 m/z, AGC of 3e6 and maximum injection time was set to 60 ms. MS2 resolution was set to 15,000, quadrupole isolation 1.7 m/z, AGC of 1e5, dynamic exclusion of 30 s and maximum injection time of 60 ms.

The nanoUPLC was coupled online through a nanoESI emitter (10 μm tip; New Objective; Woburn, MA, USA) to a quadrupole orbitrap mass spectrometer (Q Exactive HFX, Thermo Scientific) using a FlexIon nanospray apparatus (Proxeon). Data was acquired in data dependent acquisition (DDA) mode, using a Top10 method. MS1 resolution was set to 120,000 (at 400 m/z), mass range of 375–1650 m/z, AGC of 3e6 and maximum injection time was set to 60 ms. MS2 resolution was set to 15,000, quadrupole isolation 1.7 m/z, AGC of 1e5, dynamic exclusion of 30 s and maximum injection time of 60 ms (as described in^60^).

### Genomic and proteomic data processing and analysis RNASeq

Poly-A/T stretches and Illumina adapters were trimmed from the reads using cutadapt^61^ resulting reads shorter than 30 bp were discarded. Reads were mapped to the H. sapiens reference genome GRCh38 using STAR^62^ using Ensembel annotations (v.92) with EndToEnd option and outFilterMismatchNoverLmax was set to 0.04. Counts for each gene were quantified with htseq-count^63^, using the same gtf. Differential expression analysis was done using DESeq2^64^ with the betaPrior, cooksCutoff and independentFiltering parameters set to False. Raw P values were adjusted for multiple testing using the procedure of Benjamini and Hochberg. Genes that had absolute log2 fold change > 1 and *p* adjusted value < 0.05 were considered as differentially expressed. Bioinformatics Pipeline was run using snakemake^65^. All sequencing data that support the findings of this study have been deposited in the National Center for Biotechnology Information Gene Expression Omnibus (GEO) and are accessible through the GEO Series accession number GSE142314. Proteomics raw data was processed in Maxquant version 1.6.6.0. Data was searched against the SwissProt human database (November 2018 version) appended with common laboratory contaminant proteins^66^. Fixed modification was set to carbamidomethylation of cysteine and variable modifications were set to protein N-term acetylation and oxidation of methionine. Search results were filtered to achieve maximum false discovery rate of 1% at the protein level. Protein LFQ intensities were calculated based on unique peptides. The LFQ values were further processed in Perseus version 1.6.2.3. A Student’s t-test, after logarithmic transformation, was used to identify significant differences in LFQ intensities across the biological replica. The mass spectrometry proteomics data have been deposited to the ProteomeXchange Consortium via the PRIDE^67^ partner repository with the dataset identifier PXD016799. Pathway enrichment: Canonical Pathways enrichment and functional analysis were performed using Ingenuity-Pathway-Analysis (IPA) software (QIAGEN Inc., CA, US). Genes were considered differentially expressed according to the threshold of *p* value < 0.05 and fold change > 2 or < − 2.

### HT-29 human tumor xenograft in nude mice

All studies involving animals were approved by the Institutional Animal Care and Use Committee (IACUC) of the Weizmann Institute of Science (protocol # 14170519-3), all methods were performed in accordance with the relevant guidelines and regulations. Three millions of HT-29 cells in 100 µl suspension of PBS supplemented with 16.6% Matrigel (Sigma, #E6909) were inoculated subcutaneously in the right flank of animal. Tumor volumes were calculated based on the formula: tumor volume = (Length × Width × Height × 3.14/6). Following randomization into study groups (n = 5 per group), when the mean tumor size reached ~ 150 mm^3^ (6 days after tumor inoculation), animals were injected i.p. with either vehicle (saline) or indicated doses of MS023 twice/week. Animals were weighted twice/week and weight was monitored during the experiment.

### Immunohistochemistry

At 19^th^ day after tumor inoculation, mice were sacrificed and internal organs were visually examined. Tumor xenograft tissues were formalin-fixed and paraffin-embedded using standard procedures. 4 μm rehydrated samples were boiled in 10 mM citric acid solution for 10 min, blocked with 20% normal horse serum (NHS, Vector Laboratories, #S-2000) in PBS with 0.3% triton for 90 min, and avidin/biotin blocking kit (Vector Laboratories, #SP-2001) following by incubation with anti-Ki67 abs (Cell Marque, #275R-15) 1:50 overnight in RT. Then sample were treated with ABC kit (Vector Laboratories, PK-6100) for 30 min and stained with 3,3′-diaminobenzidine (DAB, Sigma, #D4293) for 3 min. Finally, cell nucleus were stained with hematoxylin by standard staining procedure. DAB images (6/sample) were randomly acquired using Nikon E600 microscope equipped with Nikon ds-Fi2 camera with both 10 × and 20 × lenses. Acquired RGB images were then splitted. The blue channel was inverted and saved in 16 bit TIF format. Image analysis was done using Metamorph “cell scoring” application, where cells expressing Ki67 were segmented with intensity threshold eightfold higher than total cells (Ki67 positive cells). Number of analyzed cell in any field was > 500.

## Supplementary information

Supplementary Figures and Tables.

## Data Availability

Data is available for review using the information below. Upon publication it will become public.* Proteomics data availability: *The mass spectrometry data has been uploaded to PRIDE (https://www.ebi.ac.uk/pride/) via ProteomeExchange. Project Name: PRMT1 inhibition induces differentiation of colon cancer cells. Project accession: PXD016799. Project DOI: Not applicable. Reviewer account details: Username: reviewer71662@ebi.ac.uk, Password: O3kMr4Sy.* Genomics data availability*: The sequencing data is available using the link below: https://www.ncbi.nlm.nih.gov/geo/query/acc.cgi?acc=GSE142314. Token: mlyjoskclrytjuf.
