# Supplementary material for: PRMT1 inhibition induces differentiation of colon cancer cells
Source: Sci Rep. 2020 Nov 18;10:20030. doi: 10.1038/s41598-020-77028-8 (PMC7676271; doi:10.1038/s41598-020-77028-8)
Supplement: Supplementary file 1 — Supplementary Figures and Tables. [file 41598_2020_77028_MOESM1_ESM.pdf]

Supplementary Data: Supplementary Tables and Figures

PRMT1 inhibition induces differentiation of colon cancer cells

Alexander Plotnikov, Noga Kozer, Galit Cohen, Silvia Carvalho, Shirly Duberstein, Ofir Almog, Leonardo Solmesky, Khriesto Shurrush, Ilana Babaev, Sima Benjamin, Shlomit Gilad, Meital Kupervaser, Yishai Levin, Michael Gershovits, Danny Ben-Avraham and Haim Michael Barr.

Content:

Supplementary Tables S1-S2

Supplementary Figures S1-S5

Supplementary Table S1. Differentially expressed proteins upon MS023 treatment.

Majority protein IDs	Gene names	Student's T-test p-value MS023 vs DMSO	Log2 Fold-Change
Q15149	PLEC	3.37E-05	-1.0883
Q7Z406	MYH14	1.71E-05	1.00307
Q5K651	SAMD9	0.00041	1.120308
Q01813	PFKP	0.000585	-1.01792
Q16822	PCK2	5.66E-05	-1.06571
P00533	EGFR	1.91E-07	-2.71627
P06737	PYGL	0.000764	1.184338
O95786	DDX58	0.006847	1.150233
Q96RL7	VPS13A	0.00016	-1.20483
Q9Y6N5	SQRDL	6.38E-06	1.032233
P08243	ASNS	9.56E-06	-1.64206
P42166	TMPO	7.21E-07	1.065289
Q13308	PTK7	0.000135	1.131121
Q08426	EHHADH	3.08E-05	-1.01181
P23786	CPT2	0.000216	-1.02867
Q8TD30	GPT2	3.49E-05	-1.39988
P47895	ALDH1A3	0.000255	1.718479
P27487	DPP4	1.97E-07	1.198329
P30519	HMOX2	0.000972	-1.04074
Q01581	HMGCS1	0.00495	1.156436
Q8IXQ6	PARP9	0.003919	1.140018
Q2M2H8		0.000114	1.296933
Q08AF3	SLFN5	0.005094	1.008626
Q05469	LIPE	0.000882	-1.20838

P22676	CALB2	0.003636	-1.37895
Q14185	DOCK1	1.26E-05	-4.00789
P09914	IFIT1	0.0025	1.179449
Q13884	SNTB1	7.10E-05	-1.04097
P98196	ATP11A	3.16E-06	-1.69475
P21589	NT5E	1.87E-07	-1.9221
Q09328	MGAT5	1.87E-06	1.089705
P46939	UTRN	2.11E-05	1.048139
Q8N122	RPTOR	2.56E-05	-2.26255
CON__P08779	KRT16	0.042993	1.067992
Q4G176	ACSF3	4.34E-05	-2.16622
Q6DKJ4	NXN	2.66E-07	-2.62726
P00966	ASS1	0.00062	1.387152
O14879	IFIT3	0.001544	1.274187
Q16836	HADH	4.68E-05	-1.51553
O43490	PROM1	7.18E-06	1.755513
Q16762	TST	3.08E-07	-1.42249
Q6UB99	ANKRD11	3.28E-05	-1.48889
P08133	ANXA6	1.66E-06	7.939053
P29728	OAS2	0.019057	1.96614
P15313	ATP6V1B1	2.56E-07	1.70622
P05362	ICAM1	2.63E-06	1.69075
P12532	CKMT1A	1.75E-05	1.498779
O75410	TACC1	4.34E-06	1.316026
P12277	CKB	0.000107	1.133894
Q9UN81	L1RE1	4.59E-05	1.062897
Q9NUI1	DECR2	4.99E-05	1.027785
Q04912	MST1R	5.51E-05	-1.03399

P09917	ALOX5	0.000118	-1.35452
O75185	ATP2C2	0.000183	-1.74371
Q0IIM8	TBC1D8B	1.44E-05	-1.84112
P28062	PSMB8	0.001221	1.433803
Q9GZM7	TINAGL1	0.00018	-1.02576
P32929	CTH	0.000192	-1.09272
Q96LB3	IFT74	0.027161	-1.09979
O95336	PGLS	0.000285	-1.51417
Q96RY7	IFT140	0.011987	-2.32387
Q9BW30	TPPP3	4.62E-05	1.983081
Q6PCE3	PGM2L1	3.33E-07	1.512651
Q6ZUJ8	PIK3AP1	4.60E-05	1.47714
P21397	MAOA	3.08E-05	1.154617
P80217	IFI35	0.000807	1.142449
Q00534	CDK6	6.76E-05	1.060429
Q8N4X5	AFAP1L2	0.000157	1.029562
Q68D91	MBLAC2	0.00097	-1.00909
Q9HBU6	ETNK1	0.000251	-1.01007
Q7Z4H8	KDELC2	0.000438	-1.03949
P35637	FUS	0.000626	-1.06407
Q16134	ETFDH	8.01E-05	-1.1747
P17252	PRKCA	6.74E-05	-1.20761
Q96GA7	SDSL	9.46E-05	1.534172
Q03518	TAP1	0.000404	1.263872
P52566	ARHGDIB	0.000854	1.212519
Q9HCH5	SYTL2	3.34E-05	1.210627
Q8WTV0	SCARB1	3.64E-05	1.194862
Q5T2W1	PDZK1;PDZK1P1	0.000766	1.088887

Q9Y4C1	KDM3A	0.000235	1.051508
Q6ZUT6	C15orf52	0.007071	1.017495
Q562E7	WDR81	0.003011	-1.02601
Q13907	IDI1	0.001502	-1.07809
Q92466	DDB2	0.001178	-1.13245
P07225	PROS1	0.000567	-1.22411
Q9NPH0	ACP6	3.08E-05	-1.27362
Q8WVT3	TRAPPC12	4.70E-05	-2.39136
Q8WZA1	POMGNT1	1.99E-05	-2.69847
A6NDB9	PALM3	2.93E-05	1.938407
Q3KQV9	UAP1L1	1.16E-06	1.610232
P15260	IFNGR1	0.00209	1.006024
Q9UHR6	ZNHIT2	0.012225	-1.08135
Q9NPE2	NGRN	0.000316	-1.10296
Q8IYS1	PM20D2	8.15E-05	-1.20856
Q6IQ22	RAB12	1.84E-06	-1.24923
P19021	PAM	0.015447	-1.71083
Q9Y5T5	USP16	0.001129	-1.88202
Q15404	RSU1	1.01E-05	-2.08432
Q8WUA7	TBC1D22A	0.000651	-2.70002
P13196	ALAS1	0.000187	-3.45057
P16455	MGMT	1.21E-05	-4.91404
Q8TAX9	GSDMB	0.00011	2.572621
Q15654	TRIP6	0.002792	1.995535
Q9H3R2	MUC13	5.31E-06	1.859414
Q86WV6	TMEM173	4.02E-05	1.383668
Q15274	QPRT	0.002134	1.079946
P15104	GLUL	0.001564	1.011682

Q8N465	D2HGDH	4.68E-07	-1.01152
Q14249	ENDOG	0.000466	-1.20194
Q9Y485	DMXL1	0.022648	-1.29066
P42126	ECI1	2.51E-05	-1.36464
Q9UPY8	MAPRE3	0.000881	-1.44235
Q6ICL3	TANGO2	0.00535	-1.49173
P48553	TRAPPC10	0.004288	-1.78483
O14640	DVL1;DVL1P1	0.001566	-1.8315
Q96EN8	MOCOS	1.38E-05	-3.3976
P10696	ALPPL2	2.41E-07	6.089202
Q8WX93	PALLD	4.00E-05	2.733094
Q13268	DHRS2	4.54E-05	2.293958
Q92597	NDRG1	2.42E-07	2.013142
P05162	LGALS2	0.000133	2.00157
P10909	CLU	3.08E-06	1.529257
Q96HN2	AHCYL2	0.012568	1.374637
O75764	TCEA3	3.70E-05	1.357185
P15428	HPGD	0.000829	1.280084
Q9BQE5	APOL2	0.000194	1.052725
Q9UKA9	PTBP2	0.00032	-1.02627
Q9H2P9	DPH5	0.002494	-1.03062
P78549	NTHL1	0.000142	-1.05835
Q5TC63	GRTP1	0.003586	-1.09233
P02750	LRG1	0.002274	-1.12611
Q9HBM1	SPC25	0.019449	-1.13134
Q9C0J9	BHLHE41	0.003891	-1.1748
P16403	HIST1H1C	0.019339	-1.43976
Q8TE76	MORC4	0.026033	-2.03954

Q16658	FSCN1	1.69E-06	3.701274
P01833	PIGR	0.004076	2.641631
P15941	MUC1	9.98E-07	2.626764
P25774	CTSS	6.64E-06	2.600855
Q9UN19	DAPP1	1.82E-05	1.966092
Q99990	VGLL1	6.48E-06	1.73875
Q9UH17	APOBEC3B	0.017737	1.689134
O95833	CLIC3	0.000182	1.535327
Q687X5	STEAP4	1.15E-05	1.522672
Q9NZU5	LMCD1	0.015808	1.214888
Q9BRX8	FAM213A	0.000486	1.170436
Q13772	NCOA4	2.03E-05	1.07237
Q96C24	SYTL4	0.002219	1.070131
P50453	SERPINB9	7.15E-05	1.026714
O43581	SYT7	0.013681	1.024292
P16444	DPEP1	7.04E-05	1.012006
Q96F63	CCDC97	0.000288	-1.03141
Q6IN84	MRM1	0.000264	-1.03777
Q9UII2	ATPIF1	0.03115	-1.1192
Q9NYB9	ABI2	3.03E-05	-1.12091
Q9Y244	POMP	0.004593	-1.14633
Q01650	SLC7A5	4.55E-05	-1.14654
Q6ZRQ5	MMS22L	0.004164	-1.16985
Q9NWZ3	IRAK4	0.002044	-1.25982
Q9NZC7	WWOX	0.021385	-1.32815
Q86SZ2	TRAPPC6B	0.000826	-1.37832
Q9Y2S2	CRYL1	0.000984	-1.45477
Q8N5V2	NGEF	5.13E-05	-1.87061

O95750	FGF19	1.48E-05	-2.14108
O60266	ADCY3	0.002075	-2.25838
P43007	SLC1A4	1.38E-06	-2.26249
P34059	GALNS	3.96E-05	-2.77069
Q02318	CYP27A1	2.31E-05	3.522551
P28065	PSMB9	0.001674	2.760162
Q01995	TAGLN	0.023138	2.209726
P53355	DAPK1	0.000306	1.758248
P18510	IL1RN	0.000166	1.556078
P09237	MMP7	0.00014	1.427749
Q8NBM8	PCYOX1L	0.002156	1.420953
Q99618	CDCA3	0.048623	1.285915
Q96EU7	C1GALT1C1	1.38E-05	1.187186
Q9NS00	C1GALT1	0.000231	1.150953
O75564	JRK	0.006977	1.07061
Q04756	HGFAC	0.001412	1.028411
P23769	GATA2	0.001105	-1.04808
A9UHW6	MIF4GD	0.006332	-1.0866
Q8N5M4	TTC9C	0.028739	-1.09526
Q9NRW3	APOBEC3C	0.007282	-1.1546
Q9NX18	SDHAF2	0.029627	-1.21972
P17676	CEBPB	0.000898	-1.30689
O75192	PEX11A	0.002512	-1.43227
Q16635	TAZ	0.003164	-1.54754
Q9HAW8	UGT1A10	0.001532	-1.64038
Q9C002	NMES1	0.032189	2.843302
Q92794	KAT6A	0.031094	2.455441
Q9BQ13	KCTD14	0.000148	1.892514

P41226	UBA7	0.043308	1.592865
Q9H190	SDCBP2	6.76E-06	1.528953
Q9UJ14	GGT7	0.000254	1.432382
Q9NQ29	LUC7L	1.28E-05	1.283855
P38571	LIPA	0.000603	1.254074
Q8N8R3	SLC25A29	0.002106	1.194402
Q9BPX7	C7orf25	0.026044	1.150054
O94929	ABLIM3	0.00171	1.123478
Q8WVN6	SECTM1	0.029054	1.100599
Q9H479	FN3K	0.043217	1.072579
Q9Y2T7	YBX2	0.001543	1.058158
Q5TF58	IFFO2	0.0027	1.048611
Q9UIS9	MBD1	0.000588	1.042593
Q9BU23	LMF2	0.001962	1.023129
Q6MZQ0	PRR5L	0.003435	1.007743
Q96RT7	TUBGCP6	0.00132	-1.06174
Q6NT76	HMBOX1	0.000599	-1.06664
A2RUS2	DENND3	0.025108	-1.12593
Q96B21	TMEM45B	0.000218	-1.15381
P51689	ARSD	3.91E-05	-1.15837
Q9Y5X9	LIPG	0.000191	-1.16673
Q9H008	LHPP	0.030402	-1.24998
Q53GA4	PHLDA2	0.003041	-1.25586
P43005	SLC1A1	0.000213	-1.26397
P07992	ERCC1	0.011749	-1.40502
Q14061	COX17	0.013884	-1.44824
Q9P0P0	RNF181	0.001978	-1.71
Q9Y6H1	CHCHD2;CHCHD2P9	0.014746	-1.72484

O75387	SLC43A1	0.004689	-1.91035
Q9UPY5	SLC7A11	0.016564	-2.1299
Q9NPA3	MID1IP1	0.000123	-2.31489
Q9HAW9	UGT1A8;UGT1A9	1.39E-06	-2.36238
Q8TB36	GDAP1	2.76E-06	2.732132
P32320	CDA	0.009199	2.266802
Q09327	MGAT3	0.001358	1.820891
Q16610	ECM1	0.00194	1.588109
P30455	HLA-A	0.000515	1.462535
P40199	CEACAM6	3.57E-05	1.437772
P34913	EPHX2	0.001126	1.294551
Q9NUL5	C19orf66	0.046884	1.231087
Q8NE22	SETD9	0.011483	1.212756
Q9Y5N5	N6AMT1	6.03E-05	1.181294
P36897	TGFBR1	0.000146	1.08646
O43521	BCL2L11	0.03838	1.066595
Q9P0T7	TMEM9	0.000918	-1.03632
P58004	SESN2	0.000446	-1.04275
Q6QHC5	DEGS2	0.000189	-1.06184
P16298	PPP3CB	3.26E-05	-1.20329
Q13303	KCNAB2	0.003721	-1.28033
Q5VT97	SYDE2	0.010723	-1.28895
Q9C005	DPY30	0.01538	-1.42467
A5PLL7	TMEM189	0.00301	-1.44263
P05386	RPLP1	0.028912	-1.55095
P54253	ATXN1	0.027126	-1.82812
Q9NYJ1	COA4	0.04954	-1.98405
P05187	ALPP	0.000104	4.363481

P40305	IFI27	0.005768	2.308998
Q14002	CEACAM7	2.22E-05	2.107024
P52895	AKR1C2	0.000147	1.649187
Q09428	ABCC8	0.011217	1.631453
P17483	HOXB4	0.002053	1.425275
Q14894	CRYM	0.002089	1.268334
P43121	MCAM	0.007952	1.261055
P06731	CEACAM5	1.43E-05	1.151409
Q99666	RGPD5	0.021693	1.033754
Q659C4	LARP1B	0.010224	-1.0211
Q8TEB9	RHBDD1	0.025951	-1.04369
Q5TA31	RNF187	0.001208	-1.0765
Q9UHJ9	PGAP2	0.000608	-1.11316
Q8NCR9	CLRN3	0.014924	-1.13773
Q9H0F6	SHARPIN	0.04698	-1.15541
P29033	GJB2	0.005155	-1.20085
A6NHC0	CAPN8	0.005491	-1.3444
Q5BKX6	SLC45A4	0.013754	-1.40576
P30989	NTSR1	0.003588	-1.45545
P57057	SLC37A1	0.001238	-1.68363
Q8N1S5	SLC39A11	2.74E-07	-1.88874
Q6P6B7	ANKRD16	0.028942	-1.90159
Q9NQ40	SLC52A3	0.010368	-1.95555
P29972	AQP1	0.008052	2.055908
Q9BT76	UPK3B	8.46E-05	1.454347
P17693	HLA-G	4.68E-05	1.355182
Q9UM01	SLC7A7	0.026292	1.23259
Q14802	FXYD3	0.01062	1.114962

Q86W74	ANKRD46	0.02336	1.097963
Q9Y3P4	RHBDD3	0.034426	1.047023
Q8IUF1	CBWD2	0.02778	-1.6146
P49450	CENPA	0.002602	-2.29674
Q9P003	CNIH4	0.000266	-3.46238

Supplementary Table S2. Expression of proteins associated with specific pathways.

Categories	Diseases or Functions Annotation	p-value	Activation z-score	Molecules	# Molecules
Cancer, Cellular Development, Cellular Growth and Proliferation, Organismal Injury and Abnormalities, Tumor Morphology	Proliferation of tumor cells	0.00894	-2.02	AFAP1L2, ALOX5, CDK6, COX17, EGFR, FGF19, HOXB9, HPGD, IL1RN, MCAM, MGMT, MST1R, MUC1, NDRG1, PRKCA, WWOX	16
Cancer, Cellular Movement, Organismal Injury and Abnormalities, Tumor Morphology	Invasion of tumor cells	0.0074	-1.802	CLU, DPP4, EGFR, HOXB9, KLK6, MCAM, MST1R, PALLD, TGFBR1	9
Cancer, Organismal Injury and Abnormalities	Growth of tumor	0.000436	-2.008	AFAP1L2, ALOX5, CDK6, CENPA, COX17, DAPK1, DPP4, EGFR, FGF19, HLA-G, HOXB9, HPGD, IL1RN, MAOA, MCAM, MGMT, MST1R, MUC1, NDRG1, NT5E, PRKCA, WWOX	22
Cancer, Organismal Injury and Abnormalities	Growth of malignant tumor	0.00422	-1.769	AFAP1L2, CENPA, COX17, EGFR, FGF19, HPGD, IL1RN, MAOA, MCAM, MGMT, MST1R, MUC1, NDRG1, PRKCA, WWOX	15
Cancer, Organismal Injury and Abnormalities	Growth of carcinoma	0.0167	-1.387	CENPA, COX17, EGFR, MAOA, MST1R	5

Cancer, Organismal Injury and Abnormalities	Solid tumor	0.0334	-1.907	ABCC8, ABI2, ABLIM3, ACP6, ACSF3, ADCY3, AFAP1L2, AHCYL2, AKR1C1/AKR1C2, ALAS1, ALDH1A3, ALOX5, ALPP, ANKRD11, ANKRD16, ANKRD46, ANXA6, APOBEC3B, APOL2, AQP1, ARHGDIB, ARSD, ASNS, ASS1, ATP11A, ATP2C2, ATP5IF1, ATP6V1B1, ATXN1, BCL2L11, BCORL1, BHLHE41, C15orf48, C19orf66, C1GALT1, C1GALT1C1, C7orf25, CALB2, CAPN8, CBWD2, CCDC50, CCDC97, CCDC9B, CDA, CDCA3, CDK6, CEACAM5, CEACAM6, CEACAM7, CEBPB, CENPA, CKB, CLIC3, CLRN3, CLU, COX17, COX18, CPT2, CRYL1, CRYM, CTH, CTSS, CYP27A1, D2HGDH, DAPK1, DAPP1, DDB2, DDX58, DECR2, DEGS2, DENND3, DHRS2, DMXL1, DOCK1, DPEP1, DPH5, DPP4, DPY30, DVL1, ECI1, ECM1, EGFR, EHHADH, ENDOG, EPHX2, ERCC1, ETFDH, ETNK1, FBLIM1, FGF19, FN3K, FSCN1, FUS, FXYD3, GALNS,	275

				GATA2, GATD3A/GATD3B, GDAP1, GGT7, GJB2, GLUL, GPT2, GRTP1, GSDMB, HADH, HGFAC, HIST1H1C, HLA-A, HLA-G, HMBOX1, HMGCS1, HMGCS2, HMOX2, HOXB4, HOXB9, HPGD, ICAM1, IDI1, IFFO2, IFI27, IFI35, IFIT1, IFIT3, IFNGR1, IFT140, IFT74, IL1RN, IRAK4, KAT6A, KCNAB2, KCTD14, KDELC2, KDM3A, KLK6, LARP1B, LGALS2, LHPP, LIPA, LIPE, LIPG, LMCD1, LMF2, LRG1, LUC7L, MAOA, MAPRE3, MBD1, MBLAC2, MCAM, MGAT3, MGAT5, MGMT, MIF4GD, MMP7, MMS22L, MOCOS, MORC4, MST1R, MUC1, MUC13, MYH14, N6AMT1, NCOA4, NDRG1, NGEF, NGRN, NT5E, NTHL1, NTSR1, NXN, OAS2, PALLD, PALM3, PAM, PARP9, PCK2, PCYOX1L, PDZK1, PEX11A, PFKP, PGAP2, PGLS, PGM2L1, PHLDA2, PIGR, PIK3AP1, PLEC, PM20D2, POMGNT1, POMP, PPP3CB, PRKCA, PROM1, PROS1, PRR5L, PSMB8,	

				PSMB9, PTBP2, PTK7, PYCR1, PYGL, QPRT, RAB12, RELB, RHBDD1, RHBDD3, RNF181, RNF187, RPLP1, RPTOR, RSU1, SAMD9, SCARB1, SDCBP2, SDHAF2, SDSL, SECTM1, SERPINB9, SESN2, SETD9, SHARPIN, SLC1A1, SLC1A4, SLC25A29, SLC37A1, SLC39A11, SLC43A1, SLC45A4, SLC52A3, SLC7A11, SLC7A5, SLC7A7, SLFN5, SNTB1, SPC25, STEAP4, SYDE2, SYT7, SYTL2, SYTL4, TACC1, TAGLN, TANGO2, TAP1, TAZ, TBC1D22A, TBC1D8B, TCEA3, TGFBR1, TINAGL1, TMEM173, TMEM189, TMEM45B, TMEM9, TMPO, TPPP3, TRAPPC10, TRAPPC6B, TRIP6, TST, TTC9C, TUBGCP6, TYSND1, UAP1L1, UBA7, UGT1A7 (includes others), UPK3B, USP16, UTRN, VGLL1, VPS13A, WDR81, WWOX, YBX2, ZNHIT2	
Cancer, Organismal Injury and Abnormalities, Tumor Morphology	Invasion of tumor	0.0137	-1.767	CLU, DPP4, EGFR, HOXB9, ICAM1, KLK6, MCAM, MST1R, PALLD, TGFBR1	10

Cell-To-Cell Signaling and Interaction	Adhesion of epithelial cells	0.00205	1.947	CLU, EGFR, ICAM1, KLK6, MST1R, MUC1, PIGR	7
Cell-To-Cell Signaling and Interaction	Interaction of tumor cell lines	0.00221	1.737	CEACAM5, DPP4, EGFR, FBLIM1, FGF19, HLA-A, ICAM1, LGALS2, MCAM, MGAT5, MUC1, PALLD, PRKCA, PROS1, RPTOR, SCARB1, TGFBR1, UTRN, WWOX	19
Cell-To-Cell Signaling and Interaction	Binding of tumor cell lines	0.00674	1.416	CEACAM5, DPP4, EGFR, FGF19, HLA-A, ICAM1, LGALS2, MCAM, MGAT5, MUC1, PALLD, PRKCA, PROS1, SCARB1, TGFBR1, UTRN, WWOX	17
Cell-To-Cell Signaling and Interaction, Cellular Assembly and Organization	Cell-cell contact of tumor cell lines	0.0204	1.4	FBLIM1, ICAM1, LGALS2, MGAT5, RPTOR	5
Cellular Movement	Cell movement of colorectal cancer cell lines	0.0098	-1.284	DOCK1, EGFR, FSCN1, KDM3A, MST1R, NDRG1, PRKCA, PROM1, RPTOR	9
Cellular Movement, Hair and Skin Development and Function	Cell movement of epithelial cell lines	0.00194	-1.709	CLU, DOCK1, EGFR, FSCN1, ICAM1, MAPRE3, MST1R, PROM1, RPTOR	9

Supplementary Figure S1

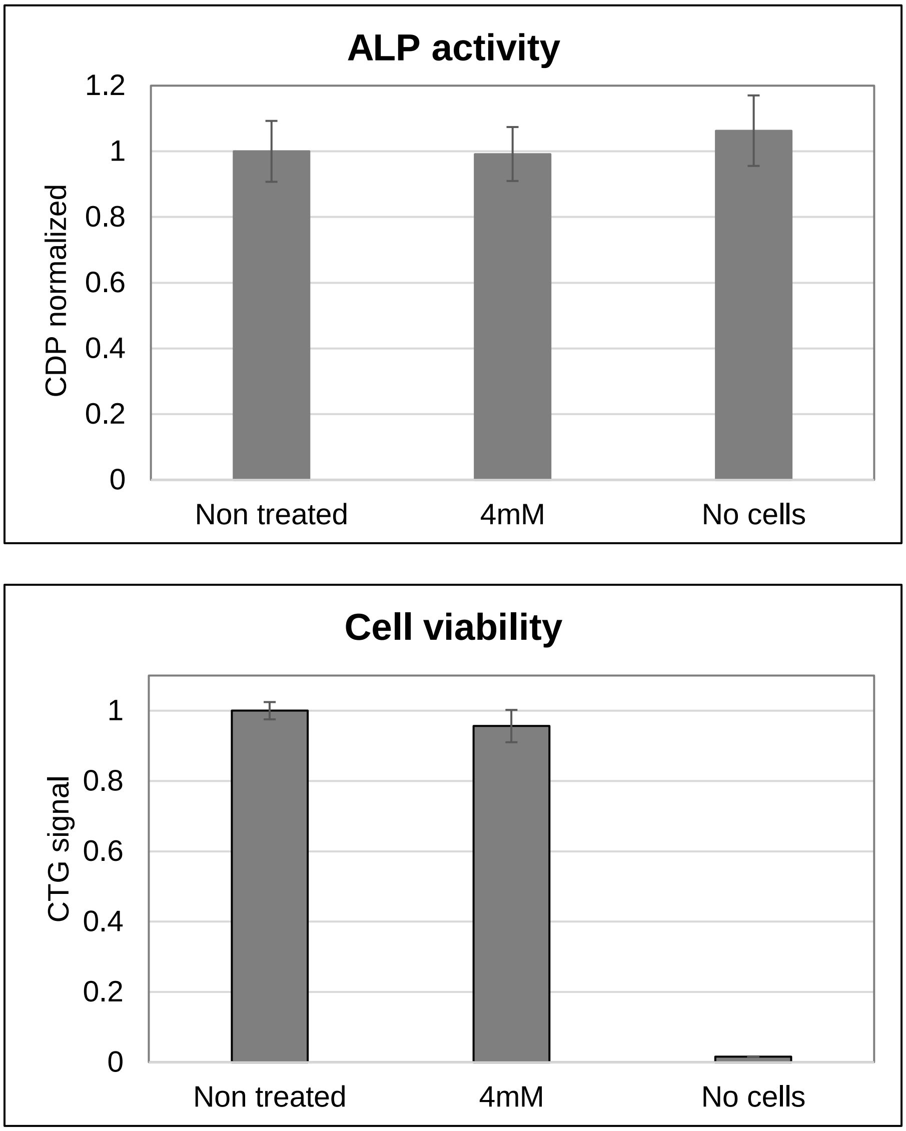

Effect of SB in the indicated concentration on colon cancer cell (HT-29) growth and ALP activity, 5 hours after treatment. Graphs represent mean $\pm$SE from three independent experiments. Statistical differences in ALP activity and cell viability between non treated and SD treated groups are not significant.

Supplementary Figure S2

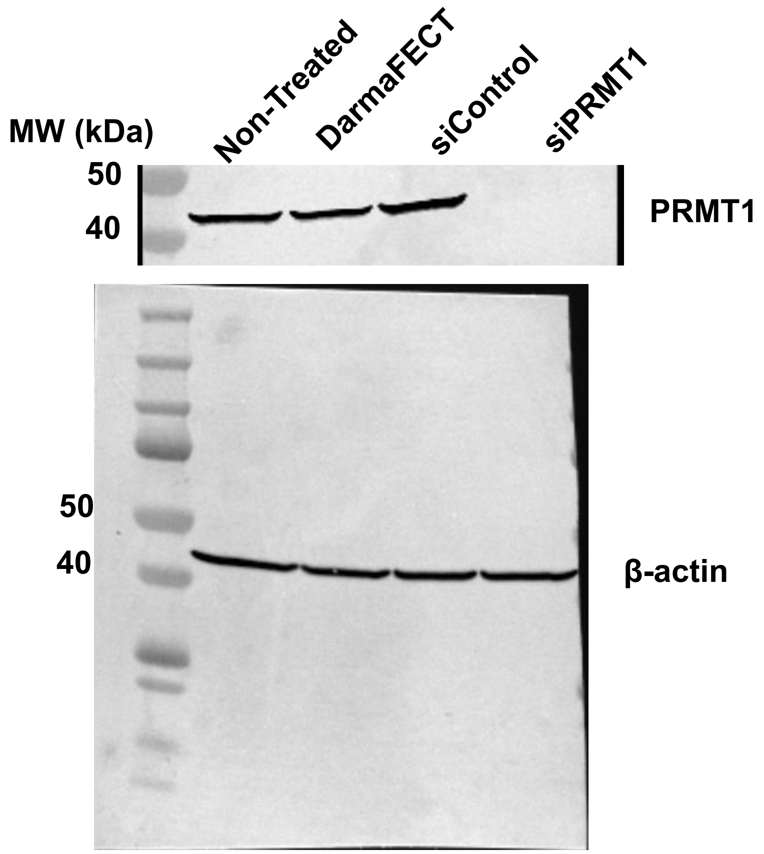

Western blot analysis of PRMT1 expression in the indicated groups of HT-29 cells. Full-length blots from Figure 3c.

Supplementary Figure S3

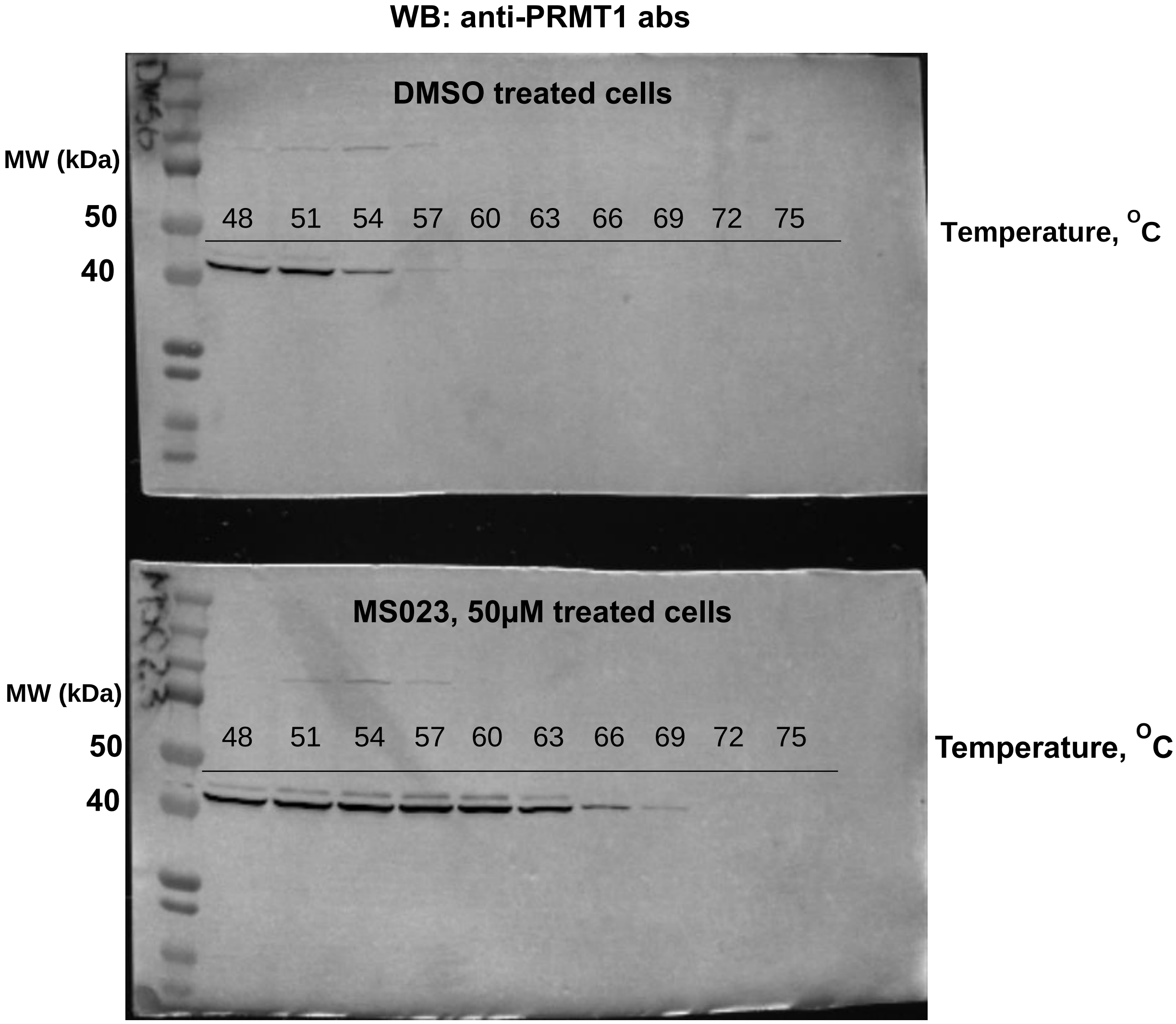

Cellular thermal shift assay of MS023. Full-length blots from Figure 4b.

Supplementary Figure S4

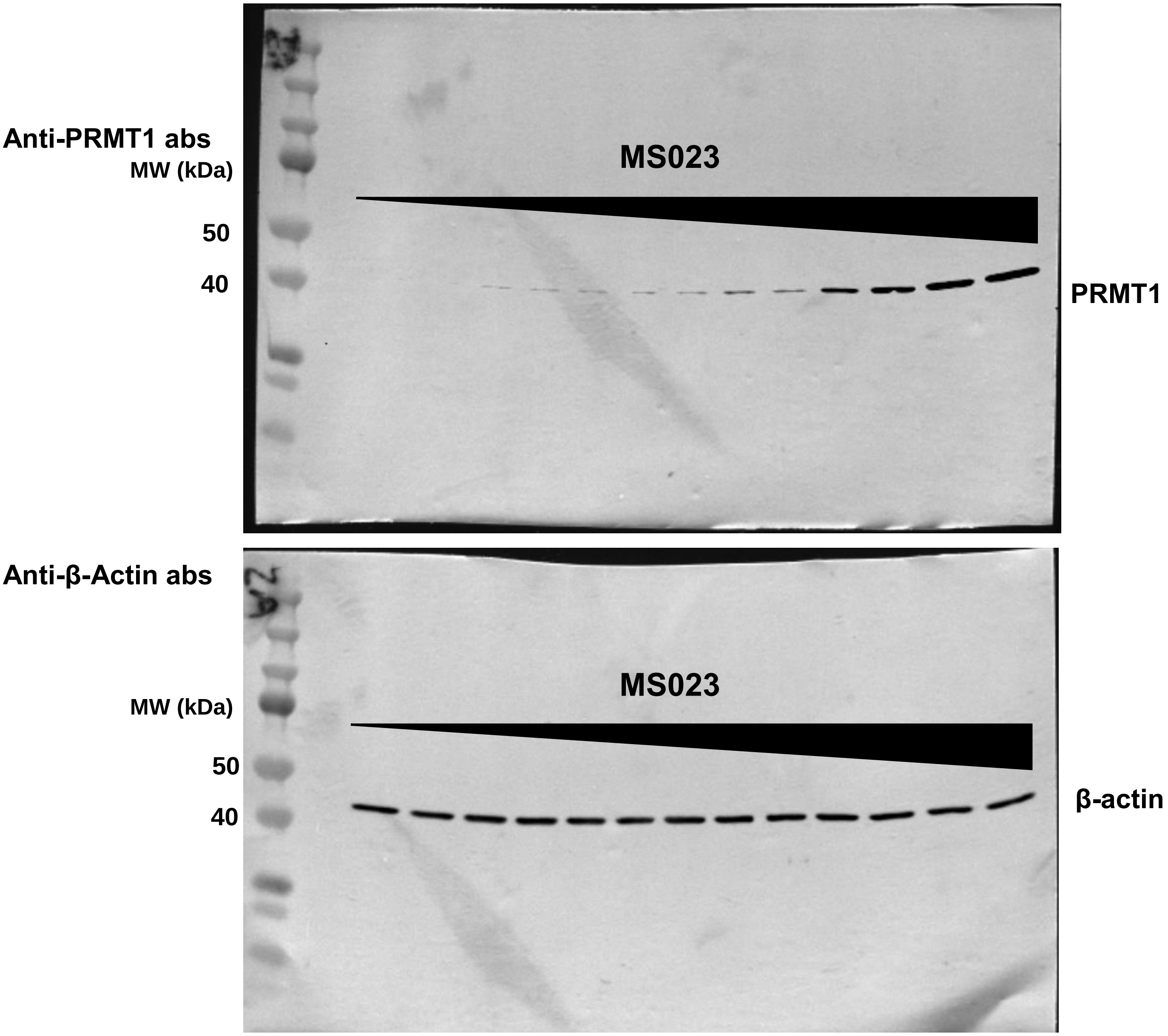

Isothermal (at 63°C) dose response fingerprints in HT-29 cells after 1h at 37°C exposure to indicated concentrations of MS023. Full-length blots from Figure 4b.

Supplementary Figure S5

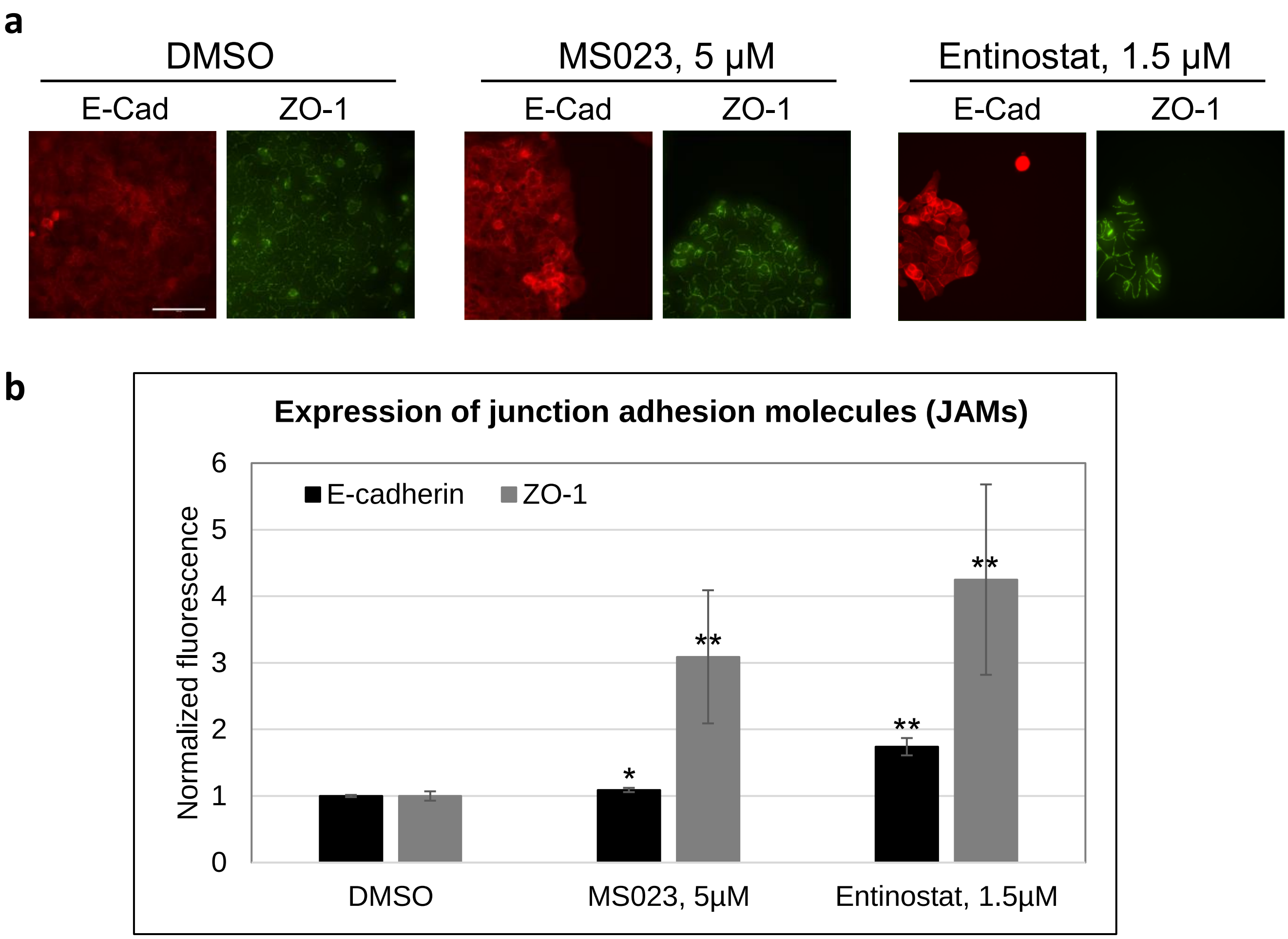

MS023 and entinostat treatment increase expression of junction adhesion molecules (JAMs) E-cadherin and ZO-1 and their genes in HCT-116 colon cancer cells. (**a**) Representative pictures of HCT-116 cells treated with indicated compounds and Graph represents mean ± SE of normalized fluorescence derived from pictures presented in "**a**". Statistical significances of JAMs induction upon MS023 and entinostat treatments are indicated.
